# Supplementary material for: The Systems Biology Research Tool: evolvable open-source software
Source: BMC Syst Biol. 2008 Jun 29;2:55. doi: 10.1186/1752-0509-2-55 (PMC2446383; doi:10.1186/1752-0509-2-55)
Supplement: Additional file 1 — SBRT Archive. An archive of the current version of the Systems Biology Research Tool. [file 1752-0509-2-55-S1.zip › sbrt-1.4.0/doc/developers_guide/api/sbrt/shell/io/AbstractMapOutputFile.html]

AbstractMapOutputFile


|  |  |  |
| --- | --- | --- |
| **Overview** \| **Package** \| **Class** \| **Use** \| **Tree** \| **Deprecated** \| **Index** \| **Help** | |  |
| **PREV CLASS**   **NEXT CLASS** | **FRAMES**    **NO FRAMES**     **All Classes** |  |
| SUMMARY: NESTED \| FIELD \| CONSTR \| METHOD | DETAIL: FIELD \| CONSTR \| METHOD |  |


---


## sbrt.shell.io Class AbstractMapOutputFile<K,V>

```
java.lang.Object
  sbrt.shell.io.AbstractMapOutputFile<K,V>
```

**Type Parameters:**: `K` - the key type.: `V` - the value type.

**All Implemented Interfaces:**: ResultsManager<java.util.Map<? extends K,? extends V>>, MapOutputFile<K,V>, MapTextFile<K,V>, MapTextOutputFile<K,V>, OutputFile<java.util.Map<? extends K,? extends V>>, SbrtFile

**Direct Known Subclasses:**: ComoFile, RomoFile

---

``` public abstract class AbstractMapOutputFile<K,V> extends java.lang.Object implements MapTextOutputFile<K,V> ```

This abstract class is a skeleton implementation of
`MapTextOutputFile`.

**Author:**
:   This class was written and documented by
    Jeremiah Wright while in the Wagner lab.

---

**Constructor Summary**

`AbstractMapOutputFile(java.lang.String fileName,
FileFormat fileFormat,
MapFormat<K,V> mapFormat)`
  
          Constructs a new vector output file from the provided
arguments.

`AbstractMapOutputFile(java.lang.String fileName,
FileFormat fileFormat,
java.util.Set<? extends K> keys,
MapFormat<K,V> mapFormat)`
  
          Constructs a new map output file from the provided
arguments.

`AbstractMapOutputFile(java.lang.String fileName,
FileFormat fileFormat,
java.lang.String allVariables,
MapFormat<K,V> mapFormat)`
  
          Constructs a new map output file from the provided
arguments.


| **Method Summary** | |
| --- | --- |
| `void` | `breakPoint()`             Writes a line of `#` symbols to this file. |
| `void` | `close()`             Closes this file. |
| `java.lang.String` | `getCanonicalName()`             Returns the canonical name of this file. |
| `UnmodifiableSet<K>` | `getFilterKeys()`             Returns the set of keys whose values will be written to this output file when a map is added. |
| `MapFormat<K,V>` | `getMapFormat()`             Returns the format used to write lines to this file. |
| `java.lang.String` | `getName()`             Returns the canonical name of this file. |
| `java.lang.String` | `getOriginalName()`             Returns the name of this file. |
| `boolean` | `isClosed()`             Indicates if this file has already been closed. |
| `protected  void` | `writeLine(java.lang.String line)`             Writes the provided string to this file as a line. |

**Methods inherited from class java.lang.Object**

`clone, equals, finalize, getClass, hashCode, notify, notifyAll, toString, wait, wait, wait`

**Methods inherited from interface sbrt.shell.io.MapOutputFile**

`addData`

**Methods inherited from interface sbrt.kernel.io.ResultsManager**

`addData`

**Constructor Detail**

### AbstractMapOutputFile

```
public AbstractMapOutputFile(java.lang.String fileName,
                             FileFormat fileFormat,
                             java.util.Set<? extends K> keys,
                             MapFormat<K,V> mapFormat)
```

:   Constructs a new map output file from the provided
    arguments.

    **Parameters:**: `fileName` - the name of the file to create.: `fileFormat` - the format of the file to create.: `keys` - the keys whose values will be written to this file. If `null`, all values from every added map will be written to this file.: `mapFormat` - the map format. **Throws:**: `java.lang.NullPointerException` - if any argument except `variables` is `null`.

---


### AbstractMapOutputFile

```
public AbstractMapOutputFile(java.lang.String fileName,
                             FileFormat fileFormat,
                             java.lang.String allVariables,
                             MapFormat<K,V> mapFormat)
```

:   Constructs a new map output file from the provided
    arguments.

    **Parameters:**: `fileName` - the name of the file to create.: `fileFormat` - the format of the file to create.: `allVariables` - a string representation of the variables whose values will be written to this file. This string is parsed by the provided vector format.: `mapFormat` - the vector format. **Throws:**: `java.lang.NullPointerException` - if any argument is `null`.

---


### AbstractMapOutputFile

```
public AbstractMapOutputFile(java.lang.String fileName,
                             FileFormat fileFormat,
                             MapFormat<K,V> mapFormat)
```

:   Constructs a new vector output file from the provided
    arguments. The value of each variable from every added
    vector will be written to this file.

    **Parameters:**: `fileName` - the name of the file to create.: `fileFormat` - the format of the file to create.: `mapFormat` - the map format. **Throws:**: `java.lang.NullPointerException` - if any argument is `null`.


**Method Detail**

### getFilterKeys

```
public UnmodifiableSet<K> getFilterKeys()
```

:   Returns the set of keys whose values will be
    written to this output file when a map is added.
    This set acts as a type of filter to control which
    values in a map are saved, and which are discarded.

    :   **Specified by:**: `getFilterKeys` in interface `MapOutputFile<K,V>`
    :   **Returns:**: the set of keys whose values will be written to this output file when a map is added; or `null` if *every* value in an added map will be written to this file.

---


### getMapFormat

```
public MapFormat<K,V> getMapFormat()
```

:   Returns the format used to write lines to this
    file.

    :   **Specified by:**: `getMapFormat` in interface `MapTextFile<K,V>`
    :   **Returns:**: the format used to write lines to this file.

---


### writeLine

```
protected final void writeLine(java.lang.String line)
```

:   Writes the provided string to this file as a line.

    :   **Parameters:**: `line` - the string to write as a line in this file.

---


### close

```
public void close()
```

:   Closes this file. If the file is already closed, no
    action will be taken.

    :   **Specified by:**: `close` in interface `ResultsManager<java.util.Map<? extends K,? extends V>>` **Specified by:**: `close` in interface `SbrtFile`

---


### getName

```
public final java.lang.String getName()
```

:   Returns the canonical name of this file.

    :   **Specified by:**: `getName` in interface `ResultsManager<java.util.Map<? extends K,? extends V>>`
    :   **Returns:**: the canonical name of this file.

---


### getOriginalName

```
public final java.lang.String getOriginalName()
```

:   Returns the name of this file.

    :   **Specified by:**: `getOriginalName` in interface `SbrtFile`
    :   **Returns:**: the name of this file.

---


### getCanonicalName

```
public final java.lang.String getCanonicalName()
```

:   Returns the canonical name of this file.

    :   **Specified by:**: `getCanonicalName` in interface `SbrtFile`
    :   **Returns:**: the canonical name of this file. **See Also:**: `File.getCanonicalPath()`

---


### isClosed

```
public boolean isClosed()
```

:   Indicates if this file has already been closed.

    :   **Specified by:**: `isClosed` in interface `ResultsManager<java.util.Map<? extends K,? extends V>>` **Specified by:**: `isClosed` in interface `SbrtFile`
    :   **Returns:**: `true` if this file is closed; `false` othewise. **See Also:**: `ResultsManager.close()`

---


### breakPoint

```
public void breakPoint()
```

:   Writes a line of `#` symbols to this file.

    :   **Specified by:**: `breakPoint` in interface `ResultsManager<java.util.Map<? extends K,? extends V>>`
    :   **Throws:**: `java.lang.IllegalStateException` - if `close()` has already been called.


---


|  |  |  |
| --- | --- | --- |
| **Overview** \| **Package** \| **Class** \| **Use** \| **Tree** \| **Deprecated** \| **Index** \| **Help** | |  |
| **PREV CLASS**   **NEXT CLASS** | **FRAMES**    **NO FRAMES**     **All Classes** |  |
| SUMMARY: NESTED \| FIELD \| CONSTR \| METHOD | DETAIL: FIELD \| CONSTR \| METHOD |  |


---
